# The Cannabis-Induced Epigenetic Regulation of Genes Associated with Major Depressive Disorder

**DOI:** 10.3390/genes13081435

**Published:** 2022-08-12

**Authors:** Guldar Sayed Mohammad, Sâmia Joca, Anna Starnawska

**Affiliations:** 1 Department of Biomedicine, Aarhus University, 8000 Aarhus, Denmark; 2 Department of BioMolecular Sciences, School of Pharmaceutical Sciences of Ribeirao Preto, University of Sao Paulo (USP), Ribeirão Preto 14040-903, Brazil; 3 The Lundbeck Foundation Initiative for Integrative Psychiatric Research, iPSYCH, 8000 Aarhus, Denmark; 4 Center for Genomics and Personalized Medicine, CGPM, Center for Integrative Sequencing, iSEQ, 8000 Aarhus, Denmark

**Keywords:** pharmacoepigenetics, DNA methylation, cannabis, cannabidiol, tetrahydrocannabinol, depression, major depressive disorder, EWAS, GWAS

## Abstract

The prevalence of depression is increasing worldwide, as is the number of people suffering from treatment-resistant depression; these patients constitute 30% of those treated. Unfortunately, there have not been significant advances in the treatment of this disorder in the past few decades. Exposure to cannabis and cannabis-derived compounds impacts depression symptomatology in different ways, with evidence indicating that cannabidiol has antidepressant effects; there have been mixed results with medical cannabis. Even though the exact molecular mechanisms of the action underlying changes in depression symptomatology upon exposure to cannabis and cannabis-derived compounds are still unknown, there is strong evidence that these agents have a widespread impact on epigenetic regulation. We hypothesized that exposure to cannabis or cannabis-derived compounds changes the DNA methylation levels of genes associated with depression. To test this hypothesis, we first performed a literature search to identify genes that are differentially methylated upon exposure to cannabis and cannabis-derived compounds, as reported in methylome-wide association studies. We next checked whether genes residing in loci associated with depression, as identified in the largest currently available genome-wide association study of depression, were reported to be epigenetically regulated by cannabis or cannabis-related compounds. Multiple genes residing in loci associated with depression were found to be epigenetically regulated by exposure to cannabis or cannabis-derived compounds. This epigenomic regulation of depression-associated genes by cannabis or cannabis-derived compounds was reported across diverse organisms, tissues, and developmental stages and occurred in genes crucial for neuronal development, functioning, survival, and synapse functioning, as well as in genes previously implicated in other mental disorders.

## 1. Introduction

Depression is a severe and debilitating mental disorder characterized by a variety of symptoms, including low mood, fatigue, decline in cognitive functioning, loss of interest and pleasure, inappropriate guilt, sleep and endocrine disturbances, and changes in appetite and psychomotor activity [1]. Depression is the most commonly diagnosed mental disorder and is estimated to be the leading global cause of years lost due to disability [2]. The lifetime prevalence of depression reaches 14%, and the risk of developing the disorder during an individual’s lifespan is influenced by both genetic and environmental factors [3,4,5,6,7,8,9,10,11,12,13]. According to twin studies, ~40% of the variation in depression liability can be attributed to additive genetic effects, and heritability estimates for changes in depression symptomatology reach 30% [14,15,16]. Recent advancements in genomic studies of depression, performed in hundreds of thousands of depression cases and unaffected controls sampled across multiple populations, confirmed that common genetic variation contributes to the development of the disorder and successfully pinpointed multiple loci across the genome associated with an increased risk of developing the condition [17,18]. The most recent and largest genome-wide association study (GWAS) of depression, performed in >1.2 million individuals, identified and replicated 192 SNPs associated with the disorder [18]. These depression-associated genetic loci were highly significantly enriched in pathways crucial for healthy brain development and functioning, including synapse assembly, organization and signaling, and the generation and differentiation of neurons [18]. As the inflection point for the discovery of genome-wide loci significant for depression was passed, it is expected that the number of discovered novel loci associated with the disorder will linearly increase with continuously increasing sample sizes [19,20]. Therefore, it is already time to start exploring how these genetic findings can have clinical applicability for patients and how they can be relevant for the pharmacological treatment of depression. 

Currently, the most standard course of depression treatment consists of a combination of psychotherapy and pharmacological treatment, with the aim of reducing depressive symptoms. Among the most commonly prescribed classes of antidepressant drugs are selective serotonin re-uptake inhibitors (SSRIs), selective serotonin noradrenaline re-uptake inhibitors (SNRIs), and tricyclic antidepressants (TCAs). Other compounds are also prescribed, such as monoamine oxidase (MAO) inhibitors, adrenergic α-2 receptor antagonists, selective noradrenaline re-uptake inhibitors, melatonin receptor agonists, serotonin 5-HT2C receptor antagonists, and selective noradrenaline/dopamine re-uptake inhibitors [21]. 

Despite the fact that pharmacological treatment for depression is widely applied and available, a meta-analysis of 165 placebo-controlled trials indicated that only 54% of adults show improvement, defined as a 50% reduction in symptoms, after weeks of treatment with antidepressant medication [21,22]. Additionally, ~30% of major depressive disorder (MDD) patients are reported to suffer from treatment-resistant depression, which is defined as depression that does not achieve full remission despite treatment with at least two different agents at adequate doses and for adequate durations [23,24]. Furthermore, 25–40% of patients who recover from MDD after treatment are reported to have another depressive episode within 2 years, 60% after 5 years, and 85% after 15 years [25,26]; altogether, these data highlight the urgent demand for the inclusion of new therapeutical compounds in the pharmacological treatment of depression. 

The cannabis plant (*Cannabis sativa*) has been used for medical and recreational purposes for thousands of years, but attention has only recently been drawn to the research of pharmacological applications of its compounds for the medical treatment of neuropsychiatric disorders [27]. Although observational studies and online surveys indicate that depression treatment is one of the main reasons why individuals make use of medical cannabis [28], nevertheless, the impairment of an individual’s overall mental health condition, including an increase in depressive symptoms, is associated with the chronic use of the plant [29,30]. In fact, the mental health impairment induced by the plant has been associated with its major psychoactive constituent, Δ9-tetrahydrocannabinol (Δ9-THC or THC) [29]. 

The cannabis plant contains more than 500 compounds, including 113 phytocannabinoids, of which Δ9-THC and cannabidiol (CBD) are the most abundant [27,31]. The compound Δ9-THC is a partial agonist at the CB1 and CB2 receptors, with well-described rewarding, analgesic, and anti-inflammatory effects [32]. While CB1 receptor activation is responsible for the psychoactive effects of THC, CB2 activation by THC seems to mediate opposite effects [33]. Unlike Δ9-THC, CBD is the primary non-psychomimetic compound found in cannabis and has a very low affinity for CB1 and CB2 receptors, often being described as a negative allosteric modulator at such targets [27]. Because of its non-psychostimulant effects, the therapeutic potential of CBD has been explored in a wide range of neuropsychiatric disorders, such as schizophrenia, epilepsy, depression, and bipolar disorder [27]. CBD was shown to reduce psychotic symptoms in schizophrenia patients and decrease anxiety in preclinical and clinical studies, and preclinical evidence suggests it to be a promising new antidepressant [34]. However, the exact molecular mechanism of the action underlying changes in these symptoms is still unknown.

**Figure 1:**
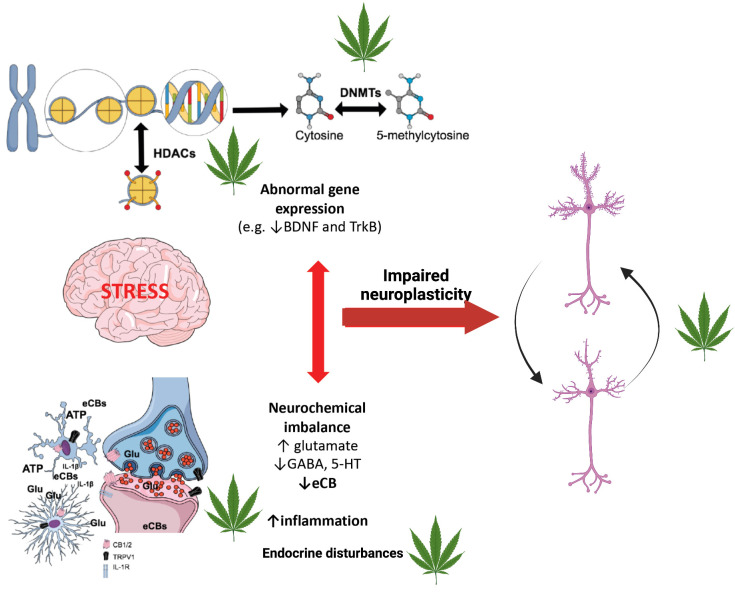
Overview of cannabis interactions with epigenetic mechanisms in relation to molecular alterations caused by stress. Stress is the main environmental factor increasing vulnerability to depression by causing dysregulation of several neurotransmitter systems (monoamines, endocannabinoids, glutamate, GABA), increased neuroinflammation, endocrine disturbances and impaired neuroplasticity, which result in impaired adaptation to subsequent aversive life events and depressed mood. All these mechanisms can be regulated by epigenetic changes, such as histone modifications (e.g. by HDACs), and DNA methylation (by DNMTs) since they exert transcriptional control over synthesizing and degrading enzymes, transporters, receptors, neurotrophins, synaptic proteins and inflammation mediators. Conversely, since the epigenetic machinery is regulated by neuronal activity, it can be directly influenced by brain’s neurochemical milleu. Cannabis, through its major constituents (CBD and THC), can “erase” stress-induced epigenetic changes by targeting neurotransmitter receptors and the neurochemical milleu, or by directly regulating the activity of DNMTs and other enzymes.

Recent research has reported that CBD modulates DNA methylation (DNAm) patterns in brain regions relevant to depression neurobiology and suggested that this epigenetic mechanism could be responsible for CBD-induced antidepressant effects [35]. An overview of cannabis interactions with epigenetic mechanisms in relation to molecular alterations caused by stress, which is a major environmental risk factor for depression, is provided in Figure 1.

DNAm is the best-studied epigenetic modification that plays a pivotal role in the regulation of neuronal development, neuronal differentiation, proper brain functioning, and, thereby, mental health. Treatments for many common mental disorders already rely on pharmaco-epigenetic treatments with therapeutic compounds that impact the expression of DNMTs and TETs (enzymes responsible for the addition and removal of methyl groups from DNA across the genome), with valproate being a prime example of such a therapeutic compound [36]. Moreover, previous evidence has demonstrated that antidepressant treatment regulates DNAm in many genes that are relevant for the neurobiology of the disease [37]. In addition, the direct pharmacological modulation of DNAm can trigger antidepressant-like effects in different animal models [38].

In this review, we hypothesized that exposure to cannabis or cannabis-derived compounds changes DNAm levels in genes associated with depression. Identification of such genes could point to an epigenetic molecular mechanism through which exposure to cannabis or cannabis-derived compounds could modify the severity of depressive symptoms and, thus, help in building a translational bridge between basic research on genetic findings for depression and the clinical practice of its treatment with novel or repurposed therapeutic compounds.

## 2. Methods

In order to answer the question of whether exposure to cannabis or cannabis-derived compounds alters DNAm patterns for genes associated with depression, the PubMed database was searched to identify relevant scientific literature by using the following combination of broad search terms: (Cannabis [Title/Abstract]) AND (DNA methylation [Title/Abstract]). In total, forty-five articles were identified according to these search criteria, with forty-four available in the English language for this study. Only primary research papers were included in this review; thus, fifteen articles identified as reviews or letters were excluded. The remaining twenty-nine articles were then filtered based on their title and abstract to include studies fulfilling four additional criteria: (i) organism-based inclusion (keeping only studies performed in human participants or animal models), (ii) tissue-based inclusion (keeping studies that measured DNAm patterns in the brain, blood, or germ line cells), (iii) genome-coverage-based inclusion (keeping studies that measured DNAm levels across the genome and excluding candidate gene studies), and (iv) relevance to the topic (keeping only studies that directly tested the association between cannabis or cannabis-derived compound exposure and differential DNAm). In total, eight epigenome-wide studies fulfilled the inclusion criteria, and the genes reported as differentially methylated in said studies were used in this review.

Genes associated with depression were identified through the most recent and largest meta-GWAS of MDD [18]. The study identified over 200 SNPs, and 192 of them were available and replicated for their association with MDD in an independent 23andMe cohort of 1,342,778 participants [18]. With the use of the FUMA tool according to the default mapping parameters, we further mapped the replicated 192 SNPs to genomic loci containing 354 genes [39].

The analysis of the overlap between the genes differentially methylated upon exposure to cannabis or cannabis-derived compounds and genes residing in loci associated with MDD was performed in the R environment [40].

## 3. Results

Eight primary research reports were identified that tested the association between cannabis or cannabis-derived compounds and changes in DNAm levels across the genome. The studies were performed in either human- (n = 5), mouse- (n = 2), or rat-derived (n = 2) tissues (one study tested DNAm changes in both human and rat samples). The tissues studied in human subjects were blood (n = 3) and sperm (n = 2), while studies performed in animal models were performed either in relevant brain regions (n = 3), such as the hippocampus, cortex, nucleus accumbens, or in sperm (n = 1). DNAm patterns were quantified with the use of either sequencing-based or DNAm array-based methods. Despite the large heterogeneity in study designs across the reviewed studies, all of them reported significant DNAm changes upon exposure to cannabis or cannabis-derived compounds [41,42,43,44,45,46,47,48]. A detailed overview of the organisms, tissues, and DNAm quantification methods used, as well as the compound exposure evaluated in each study, is presented in Table 1.

### 3.1. Epigenomic Studies in Animal Models

A 2020 study performed in mice by Wanner and co-authors reported an association between CBD exposure and DNAm changes in the adult hippocampus [41]. The study identified 3323 significantly differentially methylated positions (DMPs) and two differentially methylated regions (DMRs) between the exposed and unexposed groups, with 44% of the DMPs reported as hypermethylated and 61% of DMPs mapping to gene bodies.

A pathway analysis of the genes annotated to DMPs identified significant enrichment in pathways related to dendritic spine development, cell adhesion and migration, and excitatory postsynaptic potential. The identified genes were also overrepresented in gene sets associated with autism spectrum disorder and schizophrenia, among other gene sets [41]. 

A follow-up study performed by the same research group in 2021 tested the effect of developmental CBD exposure on genome-wide brain DNAm patterns [42]. Adult female mice (F0) were exposed to 20 mg/kg CBD daily for 9 weeks, from two weeks prior to mating through gestation and lactation, resulting in their offspring (F1) being subjected to CBD in utero and during lactation. In F0 cortex tissue, 1680 DMPs were associated with CBD exposure, in contrast to 1514 DMPs for F1 in the same tissue. CBD-induced DNAm changes identified in the cortex mapped to 266 unique genes that overlapped between F0 and F1. A total of 2012 DMPs were associated with developmental CBD exposure in the hippocampal tissue of F1. While both hyper- and hypomethylation were observed at the identified DMPs, hypomethylation was more predominant in both directly and developmentally exposed mice [42]. The genes mapped to the DMPs associated with CBD exposure, both directly in F0 and developmentally in F1, were significantly enriched in pathways highly relevant for mental health phenotypes, including neuron generation, neuron differentiation, and synaptic transmission, as well as sets of genes associated with autism spectrum disorder, epilepsy, and intellectual disability [42]. 

In addition to the animal studies testing the developmental impact of cannabis-derived compounds on DNAm changes in brain tissue, one study researched the impact of THC rather than CBD. Adolescent male and female rats were administered THC or vehicle from postnatal day 28 to postnatal day 49 and were mated as adults after the treatment was stopped and THC was no longer detected. Offspring were raised by drug-naïve mothers, and genome-wide DNAm profiles in the nucleus accumbens were investigated when F1 reached adulthood. The study reported 1027 DMRs in F1 that were associated with the adolescent THC exposure of F0. The genes that mapped to DMRs in the nucleus accumbens were significantly enriched with respect to synapse organization, regulation of membrane potential, and adult behavior pathways, among other examples [43].

### 3.2. Epigenomic Studies in Human Subjects

Along the developmental line of research regarding the effect of cannabis and cannabis-derived compounds on transgenerational epigenetic regulation, two studies investigated the impact on DNAm patterns in sperm. The first study compared the DNAm profiles of sperm from adult cannabis users (identified by a cannabis use frequency of at least once weekly for the prior 6 months) to those of non-users. In parallel, the study subjected male rats to either vehicle or 2 mg/kg THC daily for 12 days and quantified their genome-wide methylome [47]. In human subjects, 3979 CpG sites were identified as having at least a 10% methylation difference. Differentially methylated genes in cannabis users were significantly enriched in the hippo signaling pathway, glutamatergic synapse, MAPK signaling pathway, and circadian entrainment, among other examples. Differentially methylated genes in rats exposed to THC were also enriched in the hippo signaling and MAPK signaling pathways. There were several differentially methylated genes common to both humans and rats in the same enriched pathways, including *CACNA1A*, *CACNA2D1*, *CACNA1I*, *FGF12*, *PRKACA*, *APC2*, *TCFL1*, *GNB2*, *GNG7*, *BNP6*, *BNP7*, and *LLGL1*. To identify, for this review, any overlap between differentially methylated genes in the above-mentioned study and genes residing in MDD-associated loci, we used the 46 genes reported in the study that had ≥10 differentially methylated CpG sites [47]. Another study that compared DNAm differences in sperm between cannabis users and non-users identified 163 DMPs mapped to genes that enriched neurodevelopment- and cardiogenesis-related pathways [48]. Interestingly, many of the identified differences in DNAm associated with sustained cannabis use were diminished after a 77-day period of cannabis abstinence. The study also replicated findings from the previous report on genes differentially methylated in sperm due to cannabis use to be enriched in hippo signaling, glutamatergic synapse, circadian entrainment, platelet activation, mitogen-activated protein kinase signaling, and pathways in cancer [47,48].

In addition to the human studies performed in sperm, other epigenome-wide studies researched the impact of cannabis use on DNAm in blood. One of the studies reported eight DMPs in blood that were suggestively associated (*p*-value < 10^−5^) with cannabis use in individuals who were not using tobacco. Additionally, differentially methylated genes identified at a less stringent significance threshold were enriched in glutamatergic and dopaminergic synapse, long-term potentiation, and cardiomyopathy-related pathways, among others [45]. 

Another study that analyzed the impact of problematic cannabis use on blood DNAm patterns reported 543 DMPs that passed the threshold for suggestive association (*p*-value < 10^−5^), with 45 of them reaching methylome-wide significance [44]. The genes annotated to the DMPs were most significantly enriched in pathways relating to endocrine and other factor-regulated calcium reabsorption pathways, as well as cholinergic and serotonergic synapses. They were also suggestively enriched in NCAM signaling for neurite outgrowth, calcium signaling pathways, and pathways relating to cardiac conduction and cardiomyocytes [44]. The last study included in this review compared blood DNAm patterns associated with lifetime cannabis use (ever compared with never) [46]. Five DMPs were identified at a suggestive association level (*p*-value < 10^−5^), with one DMP passing the methylome-wide significance threshold. Interestingly, the study developed a blood-based multi-CpG site biomarker. The biomarker was based on 50 DMPs and was able to classify participants as lifetime cannabis users or non-users. The successful development and application of such a biomarker provide additional evidence of the association between blood DNAm and lifetime exposure to cannabis [46]. 

### 3.3. Cannabis-Associated Epigenetic Regulation of MDD Genes

**Figure 2:**
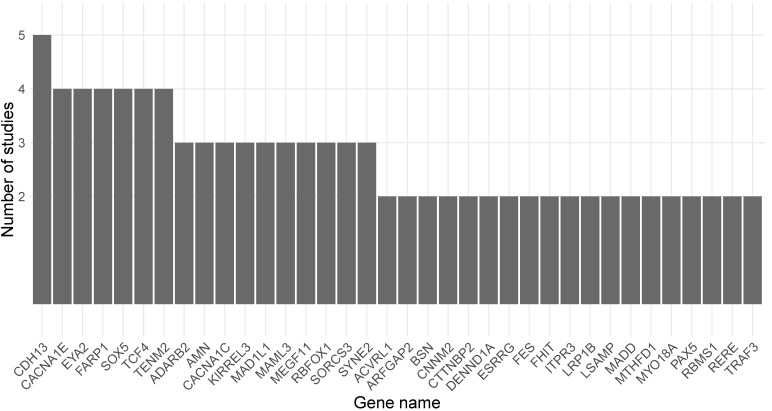
Overview of the genes overlapping between genes residing in MDD-associated loci [18] and genes identified as differentially methylated upon exposure to cannabis or cannabis-derived compounds in two or more epigenome-wide studies.

The genes mapped to DMPs or DMRs that were reported in each study as being associated with exposure to cannabis or cannabis-derived compounds were further overlapped with genes residing in MDD-associated loci [18]. An overview of the overlapping genes is presented in Table 1. Overall, 95 out of 354 genes residing in MDD-associated loci were identified as differentially methylated due to exposure to cannabis or cannabis-derived compounds, with 39 of them identified in more than one experimental study design. An overview of the number of experimental study designs in which the 39 genes were associated with exposure to cannabis or cannabis-derived compounds is provided in Figure 2. 

The most commonly identified gene overlapping with genes residing in MDD-associated loci was cadherin-13 (*CDH13*), which was reported as differentially methylated in five different study designs [41,42,43]. All of the epigenetic studies identifying *CDH13* were performed in animal models (both mouse and rat) and investigated DNAm changes in brain tissues (hippocampus, cortex, and nucleus accumbens) upon exposure to THC or CBD [41,42,43]. Additionally, eight genes (*CACNA1C*, *CACNA1E*, *EYA2*, *FARP1*, *RBFOX1*, *SOX5*, *TCF4*, and *TENM2*) residing in MDD-associated loci were identified as differentially methylated in four different study designs, and nine genes (*ADARB2*, *AMN*, *ESRRG*, *KIRREL3*, *MAD1L1*, *MAML3*, *MEGF11*, *SORCS3*, and *SYNE2*) in three different study designs (Table 1, Figure 2). Based on human studies, 12 unique genes residing in MDD-associated loci overlapped with genes differentially methylated upon exposure to cannabis [44,47,48], with 9 of them (*ESRRG*, *CACNA1C*, *GPC5*, *FAM189A1*, *MAD1L1*, *ADARB2*, *RERE*, *PCDH9*, and *RBFOX1*) also identified as overlapping with MDD genes in animal studies of exposure to CBD or THC [41,42,43]. Moreover, among the 95 genes, 5 overlapped between brain and sperm (*RERE*, *PCDH9*, *RBFOX1*, *MAD1L1*, and *ADARB2*) and 4 overlapped between blood and brain (*ESRRG*, *CACNA1C*, *GPC5*, and *FAM189A1*), indicating that there are cross-tissue epigenetic effects of cannabis and cannabis-derived compounds.

## 4. Discussion

The prevalence of depression is increasing worldwide, as is the number of patients suffering from treatment-resistant depression; these patients constitute 30% of the patient population treated for MDD [2,49,50]. In addition to the growing number of depression cases, and the burden this condition exerts on the global economy, society, and healthcare systems, there has not been significant advances in mental health treatments offered for this disorder in the past decades. These two facts highlight the urgent need for the research and identification of new therapeutic compounds that can be incorporated into depression treatment options. One of the potential new compounds recently suggested by preclinical studies as having a positive effect on alleviating depression symptoms, as well as those of anxiety and psychotic disorders, is CBD [27,34,35,51]. However, not all cannabis-derived compounds were shown to have such a positive effect. In fact, exposure to cannabis itself was associated with an increased risk of developing depression and anxiety disorders in human subjects, and it increased the prevalence of depressive- and anxiety-like behaviors in animal models [52,53]. These findings indicate that exposure to cannabis and cannabis-derived compounds impacts depression symptomatology (positively or negatively) in different ways, and detailed knowledge on the exact molecular mechanisms responsible for mediating these effects is crucial for the development of cannabinoid-based pharmacological therapies for depression-related phenotypes. 

Recent research on cannabis and cannabis-derived compounds provided strong evidence of their widespread impact on epigenetic regulation across different organisms and biological systems [54]. In this review, we explored the hypothesis that cannabis and cannabis-derived compounds (i.e., CBD and THC) change DNAm levels of genes residing in loci associated with MDD [18]. Identification of such an overlap could be a first step in understanding how cannabis and cannabis-derived compounds may, on the molecular level, contribute to changes in depression symptomatology. In this literature review, we identified 95 genes residing in MDD-associated loci that were also reported as differentially methylated upon exposure to cannabis or cannabis-derived compounds. The differential methylation of these genes was reported in both human subjects and animal models and was identified in various brain regions and peripheral tissues. The *CDH13* gene was identified most consistently among the 95 genes as differentially methylated upon exposure to cannabis-derived compounds. It acts as a negative regulator of neural proliferation, is widely expressed throughout the human brain, and is reported to increase sensitivity to drug cues [55,56,57]. *CDH13* expression is crucial for neurite outgrowth and motor neuron pathfinding, as well as synapse formation in neurons with monoaminergic or GABAergic specifications [58,59,60,61]. In addition to being associated with MDD, genetic polymorphism in *CDH13* was also linked with other mental health phenotypes, including substance abuse, smoking cessation outcomes, ADHD, violent behavior, schizophrenia, and bipolar disorder symptoms [62,63,64,65,66,67]. Moreover, *CDH13* was found to be upregulated in the amygdala of depression patients and, in parallel, was shown to play a protective role in various models exposed to environmental stressors [60,68,69,70]. Based on this evidence, it is thus conceivable that the epigenetic regulation of *CHD13* by cannabis-derived compounds could modify depression symptoms by the alteration of its expression levels, which are already known to be disturbed in the brains of depression patients [70]. 

Other genes among the 95 were consistently identified across the reviewed epigenome-wide studies, including two genes from the voltage-gated calcium channels (VGCCs) *CACNA1C* and *CACNA1E*. VGCCs are transmembrane proteins activated in response to the depolarization of the cell membrane, and they mediate the flux of calcium ions into excitable cells [71,72]. These genes are highly associated with various mental disorder phenotypes, such as neurodevelopmental disorders, intellectual disability, bipolar disorder, anxiety, and depression [73,74], and there is increasing evidence that *CACNA1C* expression in the brain directly regulates depression-related behaviors [72]. Interestingly, the genetic risk variants of mental disorders in *CACNA1C* act as both mQTLs and eQTLs; therefore, the genotype at these risk SNPs is associated with a variation in DNAm and expression levels of the gene [56,75]. It is not known whether exposure to cannabis or cannabis-derived compounds restores the DNAm and expression levels of *CACNA1C* to those of non-carriers or whether the restoration is associated with an improvement in depression symptoms; these questions need further investigation. 

*TENM2* and *TCF4* also showed changes in DNAm levels in brain tissue in animal models exposed to CBD. In addition, DMPs in sperm that varied between cannabis users and controls before and after abstinence were identified in the *TENM2* and *TCF4* genes [48]. The *TENM2* gene, also known as latrophilin-1-associated synaptic surface organizer (Lasso), is located postsynaptic in interaction, with a presynaptic latrophilin-1 in the synaptic cleft [76]. Latrophilin-1 is an adhesion G-protein-coupled receptor that contributes to the regulation of neurotransmitter release. Furthermore, the synaptic interaction of Lasso-latrophilin-1 promotes synapse formation and calcium signaling, both mechanisms especially important for neuronal development and signaling [76,77,78]. On the other hand, the *TCF4* gene encodes transcription factor 4, which is expressed during neural development. Apart from MDD, *TCF4* has also been associated with other psychiatric and neurological disorders, such as bipolar disorder and post-traumatic stress disorder [79,80]. Since the *TENM2* and *TCF4* genes are abundantly expressed in the central nervous system and especially in brain regions important for depression-related phenotypes, such as the hippocampus and cortex [56,76,81], DNAm changes in those genes due to exposure to cannabis or cannabis-derived compounds could also be of importance in future treatments for MDD.

The molecular mechanisms behind such changes in DNAm are not yet understood. There is no evidence that THC or CBD can directly interfere with the DNAm machinery, such as DNMTs, despite evidence of changes in stress-induced DNAm activity [35]. However, recent in silico evidence indicates that CBD can bind to and inhibit TET1, potentially regulating DNA demethylation [82]. It is, however, possible that the changes in DNAm result from the regulation of neurotransmitter release by cannabinoids, which can indirectly regulate the activity or gene expression levels of enzymes involved with the DNAm process [83,84].

Overall, in this review, we identified multiple genes residing in MDD-associated loci that are epigenetically regulated by cannabis or cannabis-derived compounds. There are, however, a few limitations that need to be taken into consideration when interpreting our findings and the extent of the overlap of genes across studies. Even though 39 out of the 95 genes were identified as being differentially methylated upon exposure to cannabis or cannabis-derived compounds in more than one study, many genes were identified as having altered methylation patterns in only one study. This result could be due to the large variability in study designs in the reviewed epigenome-wide reports, given that no two studies performed the exact same research experiment; as a result, we could not perform the cross-replication of the reported findings. The sources of heterogeneity in the reviewed studies involved variations in the types of compounds tested, the doses of these compounds, the developmental stages at which exposure took place, the different mammalian organisms analyzed, the tissues used, the laboratory methods used to quantify DNAm patterns genome-wide, and the statistical methods applied to identify differences in DNAm levels between compared groups [41,42,43,44,45,46,47,48]. Some of the differences in experimental design also clustered between studies, e.g., the impact of cannabis use on DNAm levels was tested in human subjects only, while the effect of exposure to cannabis-derived compounds (CBD or THC) on DNAm patterns was tested only in animal models. Additionally, epigenome-wide studies performed in human study participants were performed only in easy-to-access peripheral tissues (blood and sperm), while animal-based studies commonly used various dissected brain regions (hippocampus, cortex, and nucleus accumbens). By their nature, DNAm patterns vary greatly across tissues, cell types, genetic backgrounds, sex, age, and due to environmental exposures [85,86,87,88,89]. Therefore, DNAm patterns are likely to be impacted to a different extent at different loci depending on the study design employed. Thus, these differences between experimental setups make it difficult to conclude whether the lack of overlap with some genes differentially methylated upon exposure to cannabis or cannabis-derived compounds is due to false positive findings or due to highly variable study design. In addition to the high variability in experimental designs, different studies used different thresholds for reporting significant findings. Most studies reported genes as differentially methylated if their DMPs or DMRs were significant either study-wide or genome-wide; however, other studies reported genes with *p*-value thresholds suggestive of association (a *p*-value < 10^−5^ is commonly used for epigenome-wide association studies performed with Illumina’s methylation array). Whether more overlapping differentially methylated genes can be identified across epigenome-wide studies of cannabis or cannabis-derived compounds and whether more of these differentially methylated genes can be found to overlap with genes residing in MDD-associated loci are questions that need to be tested further by performing large, well-designed studies of these exposures across multiple independent cohorts. It is also of high interest to highlight the fact that none of the studies identified DNAm changes in cannabinoid receptors. This finding emphasizes the importance of mapping not only the targets of pharmacological compounds developed and used for the treatment of complex disorders but also researching impact of these therapeutics on epigenomic and transcriptomic regulation to gain in-depth knowledge of the influence of these agents on biological systems. 

## 5. Conclusions

Multiple genes residing in MDD-associated genomic loci are epigenetically regulated by exposure to cannabis or cannabis-derived compounds. This epigenomic regulation was reported across diverse organisms, tissues, and developmental stages, and the observed changes to DNAm occurred in genes crucial for neuronal development, functioning, survival, and synapse functioning, as well as in genes previously implicated in other mental disorders. These findings are a promising step towards understanding the epigenetic-based therapeutic potential of cannabinoids for the treatment of depression. Further molecular studies on the epigenomics, transcriptomics, and proteomics of CBD-induced changes across tissues in animal models, combined with clinical trials, are necessary to (i) evaluate whether the inclusion of CBD in the treatment of depression benefits patients and (ii) map which exact molecular changes underlie these phenotypic changes.

## Figures and Tables

**Table 1:** Overview of genes residing in GWAS MDD-associated loci [18] and genes differentially methylated upon exposure to cannabis or cannabis-derived compounds (CBD or THC).

Reference	Organism	Tissue	Exposure	DNAm Quantification Method	Genes Overlapping with MDD-Associated Loci
[32]	Mouse	HPC	20 mg/kg CBD daily for 2 weeks.	RRBS	*ELAVL4*, *NEGR1*, *CACNA1E*, *CRB1*, *GALNT2*, *TGM4*, *LSAMP*, *MAML3*, *PCDHA8*, *TENM2*, *CTTNBP2*, *PAX5*, *PHF2*, *PTCH1*, *DENND1A*, *COMTD1*, *TRIM8*, *WBP1L*, *SORCS3*, *PRRG4*, *ARFGAP2*, *NCAM1*, *KIRREL3*, *CACNA1C*, *SOX5*, *FARP1*, *SYNE2*, *DLST*, *AMN*, *HERC1*, *MEGF11*, *FES*, *RBFOX1*, *CDH13*, *TCF4*, *EYA2*
[33]	Mouse	F0 cortex	F0: Adult female mice exposed to 0 mg/kg CBD daily for 9 weeks.	RRBS	*RERE*, *CACNA1E*, *DENND1B*, *LRP1B*, *RBMS1*, *FHIT*, *LSAMP*, *NLGN1*, *MAML3*, *ADCY2*, *PCDHA1*, *PCDHA5*, *TENM2*, *MAD1L1*, *PAX5*, *DENND1A*, *CNNM2*, *MADD*, *MYBPC3*, *SPI1*, *FADS2*, *CACNA1C*, *ACVRL1*, *UNC119B*, *SPPL3*, *FARP1*, *MTHFD1*, *KLC1*, *FAM189A1*, *MEGF11*, *RBFOX1*, *CDH13*, *MYO18A*, *CELF4*, *TCF4*, *EYA2*, *ZMYND8*
		F1cortex	F1: exposed to CBD during gestation and lactation.		*CACNA1E*, *NRXN1*, *EFHD1*, *BSN*, *FHIT*, *PCDHA4*, *TENM2*, *ZSCAN12*, *MAD1L1*, *CTTNBP2*, *ADARB2*, *SORCS3*, *PAX6*, *KIRREL3*, *SOX5*, *GRASP*, *CABP1*, *OLFM4*, *SYNE2*, *RPS6KL1*, *AMN*, *FES*, *CDH13*, *MYO18A*, *TCF4*, *EYA2*
		F1: HPC	F1: exposed to CBD during gestation and lactation.		*CACNA1E*, *ESRRG*, *REEP1*, *LRP1B*, *RBMS1*, *BSN*, *RSRC1*, *MAML3*, *TMCO6*, *TENM2*, *ITPR3*, *PACRG*, *ADARB2*, *ARL3*, *SFXN2*, *NT5C2*, *INA*, *SORCS3*, *ARFGAP2*, *MADD*, *MYRF*, *FADS1*, *KIRREL3*, *CACNA1C*, *SOX5*, *ACVRL1*, *PCDH9*, *GPC5*, *FARP1*, *SYNE2*, *TRAF3*, *AMN*, *MEGF11*, *CD276*, *RBFOX1*, *SHISA9*, *CDH13*, *TCF4*, *EYA2*, *ARFGEF2*
[34]	Rat	F1: NAc	F0 exposed to 1.5 mg/kg THC every third day from postnatal day 28–49 and mated when no THC was detectable.	ERRBS	*ESRRG*, *ITPR3*, *PARK2*, *CNNM2*, *NR1H3*, *SOX5*, *FARP1*, *MTHFD1*, *TRAF3*, *CDH13*, *CTC1*
[38]	Human	Sperm	Cannabis users with use frequency at least once weekly in the last 6 months compared with non-users.	RRBS	*MAD1L1*, *ADARB2*
[39]	Human	Sperm	Cannabis users with a self-reported frequency of cannabis use at least once weekly over the prior 6 months compared to non-users.	WGBS	*RERE*, *PCDH9*, *RBFOX1*, *ASXL3*
[36]	Human	Blood	Regular cannabis users, consumed cannabis via smoking compared to matched controls.	EPIC array	No overlap between the genes identified at *p*-value < 10^−^^5^ and the ones residing in MDD-associated loci.
[35]	Human	Blood	Problematic cannabis users compared with non-users.	MBD-seq	*ESRRG*, *EYS*, *NKAIN2*, *CACNA1C*, *GPC5*, *FAM189A1*
[37]	Human	Blood	Lifetime cannabis use	450K array	No overlap between the genes identified at *p*-value < 10^−5^ and the ones residing in MDD-associated loci.

HPC: hippocampus; CBD: cannabidiol; RRBS: Reduced Representation Bisulfite Sequencing; NAc: Nucleus Accumbens; THC: Tetrahydrocannabinol; EERBS: Enhanced Reduced Representation Bisulfite Sequencing; WGBS: Whole Genome Bisulfite Sequencing; EPIC array: Infinium MethylationEPIC Array; MBD-seq: Methyl Binding Domain Sequencing; 450 K array: Infinium Methylation 450 K Array.

## References

[1] ICD-10 Version:2016 [Internet]. http://apps.who.int/classifications/icd10/browse/2016/en.

[2] World Health Organization (WHO) (2008). The Global Burden of Disease.

[3] Sullivan P.F., de Geus E.J.C., Willemsen G., James M.R., Smit J.H., Zandbelt T., Arolt V., Baune B.T., Blackwood D., Cichon S. (2009). Genome-wide association for major depressive disorder: A possible role for the presynaptic protein piccolo. Mol. Psychiatry.

[4] Wray N.R., Sullivan P.F. (2017). Genome-Wide Association Analyses Identify 44 Risk Variants and Refine the Genetic Architecture of Major Depression.

[5] van Uffelen J.G.Z., van Gellecum Y.R., Burton N.W., Peeters G., Heesch K.C., Brown W.J. (2013). Sitting-Time, Physical Activity, and Depressive Symptoms in Mid-Aged Women. Am. J. Prev. Med..

[6] Power R.A., Tansey K.E., Buttenschøn H.N., Cohen-Woods S., Bigdeli T., Hall L.S., Kutalik Z., Lee S.H., Ripke S., Steinberg S. (2017). Genome-wide Association for Major Depression Through Age at Onset Stratification: Major Depressive Disorder Working Group of the Psychiatric Genomics Consortium. Biological Psychiatry.

[7] Flint J., Kendler K.S. (2014). The Genetics of Major Depression. Neuron.

[8] Prince M.J., Harwood R.H., Blizard R.A., Thomas A., Mann A.H. (1997). Social support deficits, loneliness and life events as risk factors for depression in old age. Psychol. Med..

[9] Negele A., Kaufhold J., Kallenbach L., Leuzinger-Bohleber M. (2015). Childhood Trauma and Its Relation to Chronic Depression in Adulthood. Depress. Res. Treat..

[10] Roberts R.E., Kaplan G.A., Shema S.J., Strawbridge W.J. (1997). Prevalence and correlates of depression in an aging cohort: The Alameda County Study. J. Gerontol. B. Psychol. Sci. Soc. Sci..

[11] Mezuk B., Eaton W.W., Golden S.H., Ding Y. (2008). The Influence of Educational Attainment on Depression and Risk of Type 2 Diabetes. Am. J. Public Health.

[12] Coryell W., Endicott J., Keller M. (1992). Major depression in a nonclinical sample. Demographic and clinical risk factors for first onset. Arch. Gen. Psychiatry.

[13] Rao T.S.S., Asha M.R., Ramesh B.N., Rao K.S.J. (2008). Understanding nutrition, depression and mental illnesses. Indian J. Psychiatry.

[14] Farmer A., Harris T., Redman K., Sadler S., Mahmood A., McGuffin P. (2000). Cardiff Depression Study. Br. J. Psychiatry.

[15] Sullivan P.F., Neale M.C., Kendler K.S. (2000). Genetic Epidemiology of Major Depression: Review and Meta-Analysis. Am. J. Psychiatry.

[16] Petersen I., McGue M., Tan Q., Christensen K., Christiansen L. (2016). Change in Depression Symptomatology and Cognitive Function in Twins: A 10-Year Follow-Up Study. Twin Res. Hum. Genet..

[17] Wray N.R., Ripke S., Mattheisen M., Trzaskowski M., Byrne E.M., Abdellaoui A., Adams M.J., Agerbo E., Air T.M., Andlauer T.M.F. (2018). Genome-wide association analyses identify 44 risk variants and refine the genetic architecture of major depression. Nat. Genet..

[18] Levey D.F., Stein M.B., Wendt F.R., Pathak G.A., Zhou H., Aslan M., Quaden R., Harrington K.M., Nuñez Y.Z., Overstreet C. (2021). Bi-ancestral depression GWAS in the Million Veteran Program and meta-analysis in >1.2 million individuals highlight new therapeutic directions. Nat. Neurosci..

[19] Levinson D.F., Mostafavi S., Milaneschi Y., Rivera M., Ripke S., Wray N.R., Sullivan P.F. (2014). Genetic studies of major depressive disorder: Why are there no genome-wide association study findings and what can we do about it?. Biological Psychiatry.

[20] Visscher P.M., Wray N.R., Zhang Q., Sklar P., McCarthy M.I., Brown M.A., Yang J. (2017). 10 Years of GWAS Discovery: Biology, Function, and Translation. Am. J. Hum. Genet..

[21] Cipriani A., Furukawa T.A., Salanti G., Chaimani A., Atkinson L.Z., Ogawa Y., Leucht S., Ruhe H.G., Turner E.H., Higgins J.P.T. (2018). Comparative efficacy and acceptability of 21 antidepressant drugs for the acute treatment of adults with major depressive disorder: A systematic review and network meta-analysis. Lancet.

[22] Levkovitz Y., Tedeschini E., Papakostas G.I. (2011). Efficacy of Antidepressants for Dysthymia: A Meta-Analysis of Placebo-Controlled Randomized Trials. J. Clin. Psychiatry..

[23] Fava M. (2003). Diagnosis and definition of treatment-resistant depression. Biological Psychiatry.

[24] Li Q.S., Tian C., Hinds D., Agee M., Alipanahi B., Auton A., Bell R.K., Bryc K., Elson S.L., Fontanillas P. (2020). Genome-wide association studies of antidepressant class response and treatment-resistant depression. Transl. Psychiatry.

[25] Richards D. (2011). Prevalence and clinical course of depression: A review. Clin. Psychol. Rev..

[26] Blease C.R., O’neill S., Walker J., Hägglund M., Torous J. (2020). Treatment outcomes for depression: Challenges and opportunities. Lancet Psychiatry.

[27] Murillo-Rodriguez E., Pandi-Perumal S.R., Montii J.M. (2021). Cannabinoids and Neuropsychiatric Disorders.

[28] Sexton M., Cuttler C., Finnell J.S., Mischley L.K. (2016). A Cross-Sectional Survey of Medical Cannabis Users: Patterns of Use and Perceived Efficacy. Cannabis Cannabinoid Res..

[29] Hines L.A., Freeman T., Gage S.H., Zammit S., Hickman M., Cannon M., Munafo M., Macleod J., Heron J. (2020). Association of High-Potency Cannabis Use With Mental Health and Substance Use in Adolescence. JAMA Psychiatry.

[30] Memedovich K.A., Dowsett L.E., Spackman E., Noseworthy T., Clement F. (2018). The adverse health effects and harms related to marijuana use: An overview review. Can. Med. Assoc. Open Access J..

[31] Small E. (2015). Evolution and Classification of Cannabis sativa (Marijuana, Hemp) in Relation to Human Utilization. Bot. Rev..

[32] Shahbazi F., Grandi V., Banerjee A., Trant J.F. (2020). iScience Cannabinoids and Cannabinoid Receptors: The Story So Far.

[33] Li X., Hempel B.J., Yang H.-J., Han X., Bi G.-H., Gardner E.L., Xi Z.-X. (2021). Dissecting the role of CB1 and CB2 receptors in cannabinoid reward versus aversion using transgenic CB1- and CB2-knockout mice. European Neuropsychopharmacology.

[34] Joca S., Silote G.P., Sartim A., Sales A., Guimarães F., Wegener G. (2021). Putative effects of cannabidiol in depression and synaptic plasticity. The Neuroscience of Depression.

[35] Sales A.J., Guimarães F.S., Joca S.R. (2020). CBD modulates DNA methylation in the prefrontal cortex and hippocampus of mice exposed to forced swim. Behavioural Brain Research.

[36] Milutinovic S., D’Alessio A.C., Detich N., Szyf M. (2007). Valproate induces widespread epigenetic reprogramming which involves demethylation of specific genes. Carcinogenesis.

[37] Sales A.J., Guimarães F.S., Joca S.R.L. (2021). DNA methylation in stress and depression: From biomarker to therapeutics. Acta Neuropsychiatr..

[38] Sales A.J., Maciel I.S., Suavinha A.C.D.R., Joca S.R.L. (2020). Modulation of DNA Methylation and Gene Expression in Rodent Cortical Neuroplasticity Pathways Exerts Rapid Antidepressant-Like Effects. Molecular Neurobiology.

[39] Watanabe K., Taskesen E., van Bochoven A., Posthuma D. (2017). Functional mapping and annotation of genetic associations with FUMA. Nat. Commun..

[40] Team R.C.R. (2015). A Language and Environment for Statistical Computing. http://www.gbif.org/resource/81287.

[41] Wanner N.M., Colwell M., Drown C., Faulk C. (2020). Subacute cannabidiol alters genome-wide DNA methylation in adult mouse hippocampus. Environmental and Molecular Mutagenesis.

[42] Wanner N.M., Colwell M., Drown C., Faulk C. (2021). Developmental cannabidiol exposure increases anxiety and modifies genome-wide brain DNA methylation in adult female mice. Clin. Epigenet..

[43] Watson C.T., Szutorisz H., Garg P., Martin Q., Landry J.A., Sharp A.J., Hurd Y.L. (2015). Genome-Wide DNA Methylation Profiling Reveals Epigenetic Changes in the Rat Nucleus Accumbens Associated With Cross-Generational Effects of Adolescent THC Exposure. Neuropsychopharmacology.

[44] Clark S.L., Chan R., Zhao M., Xie L.Y., Copeland W.E., Aberg K.A., Oord E.J.V.D. (2021). Methylomic Investigation of Problematic Adolescent Cannabis Use and Its Negative Mental Health Consequences. Child & Adolescent Psychiatry.

[45] Osborne A.J., Pearson J.F., Noble A.J., Gemmell N.J., Horwood L.J., Boden J.M., Benton M.C., Macartney-Coxson D.P., Kennedy M.A. (2020). Genome-wide DNA methylation analysis of heavy cannabis exposure in a New Zealand longitudinal cohort. Transl. Psychiatry.

[46] Markunas C.A., Hancock D., Xu Z., Quach B.C., Fang F., Sandler D.P., Johnson E.O., Taylor J.A. (2020). Epigenome-wide analysis uncovers a blood-based DNA methylation biomarker of lifetime cannabis use. American Journal of Medical Genetics Part B: Neuropsychiatric Genetics.

[47] Acharya K., Mitchell J.T., Visco Z., Grenier C., Murphy S.K., Schrott R., Hall B.J., Price T.M., McClernon J., Levin E.D. (2020). Data from: Cannabinoid Exposure and Altered DNA Methylation in Rat and Human Sperm.

[48] Schrott R., Murphy S.K., Modliszewski J.L., King D.E., Hill B., Itchon-Ramos N., Raburn D., Price T., Levin E.D., Vandrey R. (2021). Refraining from use diminishes cannabis-associated epigenetic changes in human sperm. Environmental Epigenetics.

[49] Mojtabai R., Olfson M., Han B. (2016). National Trends in the Prevalence and Treatment of Depression in Adolescents and Young Adults. Pediatrics.

[50] Al-Harbi K.S. (2012). Treatment-resistant depression: Therapeutic trends, challenges, and future directions. Patient Preference Adherence.

[51] García-Gutiérrez M.S., Navarrete F., Gasparyan A., Austrich-Olivares A., Sala F., Manzanares J. (2020). Cannabidiol: A Potential New Alternative for the Treatment of Anxiety, Depression, and Psychotic Disorders. Biomolecules.

[52] Mustonen A., Hielscher E., Miettunen J., Denissoff A., Alakokkare A.-E., Scott J.G., Niemelä S. (2021). Adolescent cannabis use, depression and anxiety disorders in the Northern Finland Birth Cohort 1986. BJPsych Open.

[53] Mohammed A.M., Khardali I.A., Oraiby M.E., Hakami A.F., Shaheen E.S., Ageel I.M., Abutawil E.H., Abu-Taweel G.M. (2021). Anxiety, depression-like behaviors and biochemistry disorders induced by cannabis extract in female mice. Saudi J. Biol. Sci..

[54] Szutorisz H., Hurd Y.L. (2015). Epigenetic Effects of Cannabis Exposure. Biol. Psychiatry.

[55] Takeuchi T., Misaki A., Liang S.-B., Tachibana A., Hayashi N., Sonobe H., Ohtsuki Y. (2002). Expression of T-cadherin (CDH13, H-Cadherin) in human brain and its characteristics as a negative growth regulator of epidermal growth factor in neuroblastoma cells. J. Neurochem..

[56] GTEx Consortium (2013). The Genotype-Tissue Expression (GTEx) project. Nat. Genet..

[57] King C.P., Militello L., Hart A., Pierre C.L.S., Leung E., Versaggi C.L., Roberson N., Catlin J., Palmer A.A., Richards J.B. (2017). *Cdh13* and *AdipoQ* gene knockout alter instrumental and Pavlovian drug conditioning. Genes Brain Behav..

[58] Fredette B.J., Miller J., Ranscht B. (1996). Inhibition of motor axon growth by T-cadherin substrata. Development.

[59] Rivero O., Sich S., Popp S., Schmitt A., Franke B., Lesch K.-P. (2013). Impact of the ADHD-susceptibility gene CDH13 on development and function of brain networks. European Neuropsychopharmacology.

[60] Kiser D.P., Popp S., Schmitt-Böhrer A.G., Strekalova T., Hove D.L.V.D., Lesch K.-P., Rivero O. (2018). Early-life stress impairs developmental programming in Cadherin 13 (CDH13)-deficient mice. Progress in Neuro-Psychopharmacology and Biological Psychiatry.

[61] Drgonova J., Walther N., Hartstein G.L., Bukhari M.O., Baumann M., Katz J., Hall F.S., Arnold E.R., Flax S., Riley A. (2016). Cadherin 13: Human cis-Regulation and Selectively Altered Addiction Phenotypes and Cerebral Cortical Dopamine in Knockout Mice. Mol. Med..

[62] Johnson C., Drgon T., Walther N., Uhl G.R. (2011). Genomic Regions Identified by Overlapping Clusters of Nominally-Positive SNPs from Genome-Wide Studies of Alcohol and Illegal Substance Dependence. PLoS ONE.

[63] Drgon T., Montoya I., Johnson C., Liu Q.-R., Walther D., Hamer D., Uhl G.R. (2009). Genome-Wide Association for Nicotine Dependence and Smoking Cessation Success in NIH Research Volunteers. Mol. Med..

[64] Salatino-Oliveira A., Genro J.P., Polanczyk G.V., Zeni C., Schmitz M., Kieling C., Anselmi L., Menezes A.M.B., Barros F.C., Polina E.R. (2015). Cadherin-13 gene is associated with hyperactive/impulsive symptoms in attention/deficit hyperactivity disorder. Am. J. Med Genet. B Neuropsychiatr. Genet..

[65] Tiihonen J., Rautiainen M.-R., Ollila H., Repotiihonen E., Virkkunen M., Palotie A., Pietilainen O., Kristiansson K., Joukamaa M., Lauerma H. (2014). Genetic background of extreme violent behavior. Mol. Psychiatry.

[66] Børglum A.D., Demontis D., Grove J., Pallesen J., Hollegaard M.V., Pedersen C.B., Hedemand A., Mattheisen M., Uitterlinden A., Nyegaard M. (2014). Genome-wide study of association and interaction with maternal cytomegalovirus infection suggests new schizophrenia loci. Mol. Psychiatry.

[67] Cho C.-H., Lee H.-J., Woo H.G., Choi J.-H., Greenwood T.A., Kelsoe J.R. (2015). *CDH13* and *HCRTR2* May Be Associated with Hypersomnia Symptom of Bipolar Depression: A Genome-Wide Functional Enrichment Pathway Analysis. Psychiatry Investig..

[68] Denzel M.S., Scimia M.-C., Zumstein P.M., Walsh K., Ruiz-Lozano P., Ranscht B. (2010). T-cadherin is critical for adiponectin-mediated cardioprotection in mice. J. Clin. Investig..

[69] Hebbard L.W., Garlatti M., Young L.J., Cardiff R.D., Oshima R.G., Ranscht B. (2008). T-cadherin Supports Angiogenesis and Adiponectin Association with the Vasculature in a Mouse Mammary Tumor Model. Cancer Res..

[70] Sibille E., Wang Y., Joeyen-Waldorf J., Gaiteri C., Surget A., Oh S., Belzung C., Tseng G.C., Lewis D. (2009). A Molecular Signature of Depression in the Amygdala. Am. J. Psychiatry.

[71] Andrade A., Brennecke A., Mallat S., Brown J., Gomez-Rivadeneira J., Czepiel N., Londrigan L. (2019). Genetic Associations between Voltage-Gated Calcium Channels and Psychiatric Disorders. Int. J. Mol. Sci..

[72] Kabir Z.D., Lee A.S., Burgdorf C.E., Fischer D.K., Rajadhyaksha A.M., Mok E., Rizzo B., Rice R.C., Singh K., Ota K.T. (2016). Cacna1c in the Prefrontal Cortex Regulates Depression-Related Behaviors via REDD1. Neuropsychopharmacology.

[73] Moon A.L., Haan N., Wilkinson L.S., Thomas K.L., Hall J. (2018). CACNA1C: Association With Psychiatric Disorders, Behavior, and Neurogenesis. Schizophr. Bull..

[74] Royer-Bertrand B., Gygax M.J., Cisarova K., Rosenfeld J.A., Bassetti J.A., Moldovan O., O’Heir E., Burrage L.C., Allen J., Emrick L.T. (2021). De novo variants in CACNA1E found in patients with intellectual disability, developmental regression and social cognition deficit but no seizures. Mol. Autism..

[75] Starnawska A., Demontis D., Pen A., Hedemand A., Nielsen A.L., Staunstrup N.H., Grove J., Als T.D., Jarram A., O’Brien N.L. (2016). CACNA1C hypermethylation is associated with bipolar disorder. Transl. Psychiatry.

[76] Vysokov N.V., Silva J.P., Lelianova V.G., Ho C., Djamgoz M.B., Tonevitsky A.G., Ushkaryov Y.A. (2016). The Mechanism of Regulated Release of Lasso/Teneurin-2. Front. Mol. Neurosci..

[77] Vysokov N.V., Silva J.P., Lelianova V.G., Suckling J., Cassidy J., Blackburn J.K. (2018). Proteolytically released Lasso/teneurin-2 induces axonal attraction by interacting with latrophilin-1 on axonal growth cones. Elife.

[78] Silva J.-P., Lelianova V.G., Ermolyuk Y.S., Vysokov N., Hitchen P.G., Berninghausen O., Rahman M.A., Zangrandi A., Fidalgo S., Tonevitsky A.G. (2011). Latrophilin 1 and its endogenous ligand Lasso/teneurin-2 form a high-affinity transsynaptic receptor pair with signaling capabilities. Proc. Natl. Acad. Sci. USA.

[79] Teixeira J.R., Szeto R.A., Carvalho V.M.A., Muotri A.R., Papes F. (2021). Transcription factor 4 and its association with psychiatric disorders. Transl. Psychiatry.

[80] Gelernter J., Sun N., Polimanti R., Pietrzak R., Levey D., Bryois J., Lu Q., Hu Y., Li B. (2019). Genome-wide association study of post-traumatic stress disorder reexperiencing symptoms in >165,000 US veterans. Nat. Neurosci..

[81] Tessarin G.W.L., Michalec O.M., Torres-Da-Silva K.R., Da Silva A.V., Cruz-Rizzolo R.J., Gonçalves A., Gasparini D.C., Horta-Junior J.D.A., Ervolino E., Bittencourt J.C. (2019). A Putative Role of Teneurin-2 and Its Related Proteins in Astrocytes. Frontiers in Neuroscience.

[82] Antonyová V., Kejík Z., Brogyanyi T., Kaplánek R., Veselá K., Abramenko N., Ocelka T., Masařík M., Matkowski A., Gburek J. (2022). Non-psychotropic cannabinoids as inhibitors of TET1 protein. Bioorganic Chemistry.

[83] Rusconi F., Rubino T., Battaglioli E. (2020). Endocannabinoid-Epigenetic Cross-Talk: A Bridge toward Stress Coping. Int. J. Mol. Sci..

[84] Rusconi F., Battaglioli E. (2018). Acute Stress-Induced Epigenetic Modulations and Their Potential Protective Role Toward Depression. Frontiers in Molecular Neuroscience.

[85] Romanoski C.E., Glass C.K., Stunnenberg H.G., Wilson L., Almouzni G. (2015). Epigenomics: Roadmap for regulation. Nature.

[86] Consortium R.E., Kundaje A., Meuleman W., Ernst J., Bilenky M., Yen A., Heravi-Moussavi A., Kheradpour P., Zhang Z., Wang J. (2015). Integrative analysis of 111 reference human epigenomes. Nature.

[87] Smith A.K., Kilaru V., Kocak M., Almli L.M., Mercer K.B., Ressler K.J., Tylavsky F.A., Conneely K.N. (2014). Methylation quantitative trait loci (meQTLs) are consistently detected across ancestry, developmental stage, and tissue type. BMC Genom..

[88] Martin E.M., Fry R.C. (2018). Environmental Influences on the Epigenome: Exposure- Associated DNA Methylation in Human Populations. Annu. Rev. Public Health.

[89] Horvath S. (2013). DNA methylation age of human tissues and cell types. Genome Biol..

